# Biogeography of the large intestinal mucosal and luminal microbiome in cynomolgus macaques with depressive-like behavior

**DOI:** 10.1038/s41380-021-01366-w

**Published:** 2021-11-01

**Authors:** Teng Teng, Gerard Clarke, Michael Maes, Yuanliang Jiang, Jun Wang, Xuemei Li, Bangmin Yin, Yajie Xiang, Li Fan, Xueer Liu, Jie Wang, Shouhuan Liu, Yunqing Huang, Julio Licinio, Xinyu Zhou, Peng Xie

**Affiliations:** 1 Department of Neurology, The First Affiliated Hospital of Chongqing Medical University, Chongqing, China; 2 NHC Key Laboratory of Diagnosis and Treatment on Brain Functional Diseases, The First Affiliated Hospital of Chongqing Medical University, Chongqing, China; 3 Department of Psychiatry and Neurobehavioural Science, University College Cork, Cork, Ireland; 4 APC Microbiome Ireland, University College Cork, Cork, Ireland; 5 Department of Psychiatry, Faculty of Medicine, Chulalongkorn University, Bangkok, Thailand; 6 Department of Psychiatry, Medical University of Plovdiv, Plovdiv, Bulgaria; 7 School of Medicine, IMPACT Strategic Research Centre, Deakin University, Geelong, VIC Australia; 8 Department of Psychiatry, The First Affiliated Hospital of Chongqing Medical University, Chongqing, China; 9 CAS Key Laboratory of Pathogenic Microbiology and Immunology, Institute of Microbiology, Chinese Academy of Sciences, Beijing, China; 10 Shanghai Applied Protein Technology Co., Ltd, Shanghai, China; 11 Department of Psychiatry and Behavioral Sciences, College of Medicine, State University of New York (SUNY) Upstate Medical University, Syracuse, NY USA; 12 Department of Neuroscience & Physiology, College of Medicine, SUNY Upstate Medical University, Syracuse, NY USA

**Keywords:** Molecular biology, Depression

## Abstract

Most previous studies in the pathophysiology of major depressive disorder (MDD) focused on fecal samples, which limit the identification of the gut mucosal and luminal microbiome in depression. Here, we address this knowledge gap. Male cynomolgus macaques (*Macaca fascicularis*) were randomly assigned to a chronic unpredictable mild stress (CUMS) group, or to an unstressed control group. Behavioral tests were completed in both groups. At endpoint, microbe composition of paired mucosal and luminal samples from cecum, ascending, transverse, and descending colons were determined by 16S ribosomal RNA gene sequencing. The levels of 34 metabolites involved in carbohydrate or energy metabolism in luminal samples were measured by targeted metabolomics profiling. CUMS macaques demonstrated significantly more depressive-like behaviors than controls. We found differences in mucosal and luminal microbial composition between the two groups, which were characterized by Firmicutes and Bacteriodetes at the phylum level, as well as Prevotellaceae and Lachnospiraceae at the family level. The majority of discriminative microbes correlated with the depressive-like behavioral phenotype. In addition, we found 27 significantly different microbiome community functions between the two groups in mucosa, and one in lumen, which were mainly involved in carbohydrate and energy metabolism. A total of nine metabolites involved in these pathways were depleted in CUMS animals. Together, CUMS macaques with depressive-like behaviors associated with distinct alterations of covarying microbiota, carbohydrate and energy metabolism in mucosa and lumen. Further studies should focus on the mucosal and luminal microbiome to provide a deeper spatiotemporal perspective of microbial alterations in the pathogenesis of MDD.

## Introduction

Major depression disorder (MDD) currently affects over 300 million people worldwide, and it is the leading contributor to burden of mental health-related disease [1]. Currently, there are numerous neurobiological perturbations which may plausibly account for MDD symptoms, such as deficits in monoamine neurotransmitters [2], immune system alterations [3, 4], endocrine disturbances [5], and neurotrophin alterations [6]. The pathogenesis of these perturbations remains elusive. Recent findings demonstrated that abnormalities in the gut microbiome have emerged as a key component in the pathophysiology of depression [7]. We recently reported that MDD was associated with an altered gut microbiome, and the transplantation of MDD stool microbiota to germ-free mice or microbiota-depleted rats resulted in depressive-like behaviors [8, 9]. Most of previous clinical studies have focused on the fecal microbiome, without specifically addressing the gut mucosal and luminal microbiome [10, 11]. The fecal microbiome is different from the microbial communities at gut mucosal and luminal sites, and this gradient also varies spatially at different sites along the length of the colon [12]. Therefore, understanding the relationship and differences between gut mucosal and luminal microbial signatures in MDD is critical to understand the role of the gut microbiome in the pathogenesis of MDD.

It is not feasible to collect mucosal and luminal samples throughout the intestinal tract in MDD patients. Rodent models are commonly used in depression [13] and the mucosal and luminal contents of mice are readily accessible; however, neither the pelleted, sparse nature of their colonic contents nor their native microbial composition are fully representative of the human gut [14]. A recent study reported that only 5.59% of identified microbial genes overlapped between human and mice; in contrast, 39.49% of identified bacterial genes in cynomolgus macaques were found in human samples [15]. More importantly, non-human primates (NHP) offer superior mechanistic insights due to the increased similarities across neurodevelopment, social behavior, emotion regulation and stress response with humans [16]. Previously, consistent with Shively et al.’s findings [17], we have developed a system for evaluating, defining, and classifying depressive-like and other behaviors of captive cynomolgus macaques *(Macaca fascicularis*) [18]. Recently, we adapted and validated a chronic unpredictable mild stress (CUMS) protocol for use in these animals, with features reflecting the depressive-like behaviors, stressors and biologic changes observed in MDD patients [19]. By inference, the CUMS monkey paradigm may be a useful new model for studying the gut microbiota in the pathogenesis of MDD.

Here, we compared the biogeography of the large intestinal mucosal and luminal microbiome of cynomolgus CUMS macaques and healthy controls by using 16S ribosomal RNA (rRNA) gene sequencing, and calculate the relationship between the discriminative amplicon sequence variants (ASVs) and depressive-like behaviors. Then, we constructed the key covarying networks in these animals by co-occurrence analysis based on the relative abundance of discriminative ASVs. Finally, we calculated the predicted microbiome community functions in mucosa and lumen, and the metabolites in the key altered metabolism pathways were validated by targeted metabolomics profiling.

## Materials and methods

### Stress procedures and behavioral observation

The detailed behavioral paradigm and experiment protocol were reported in our previous study [19]. Briefly, ten healthy male cynomolgus macaques (*Macaca fascicularis*) aged from 1 to 4 years old were selected, paired on age and body weight, and each member of a pair was randomly assigned to either the CUMS group (CUMS, *n* = 5) or unstressed control group (CON, *n* = 5). The CUMS group was exposed to multiple unpredictable mild stressors (e.g., noise, water deprivation, fasting, cold stress, and space restriction) for five cycles (one cycle equals 7 days multiple stress plus 4 day behavior observation), and then conducted behavioral observations and tests (e.g., the attempt for apple test [AAT] and human intruder test [HIT]).

### Subjects and sample collection

The entire intestinal tract was removed from the body at euthanasia. Then, the cecum, ascending colon, transverse colon, and descending colon were each cross-sectioned, and longitudinally transected from the anti-mesenteric side of the intestine to open the intestinal lumen. The intestinal contents were removed from the lumen, and intestinal mucosal samples were scraped after rinsing with sterile saline until there were no visible contents. All the samples were stored at −80 °C for further studies. Samples were collected from mucosa and lumen, at four locations (cecum, ascending colon, transverse colon, and descending colon), from the 10 animals, resulting in 80 samples.

### 16S rRNA sequencing

We used our well established pipelines of DNA extraction, Polymerase Chain Reaction (PCR) amplification and Illumina MiSeq Sequencing as previously described [8]. In brief, we extracted microbial DNA from intestinal luminal and mucosal samples using the E.Z.N.A.^®^ soil DNA Kit (Omega Bio-tek, Norcross, GA, USA). The hypervariable region of the bacterial 16S rRNA gene (V3-V4) was amplified with primer pairs 338F (5′-ACTCCTACGGGAGGCAGCAG-3′) and 806R (5′-GGACTACHVGGGTWTCTAAT-3′) by an ABI GeneAmp^®^ 9700 PCR thermocycler (ABI, CA, USA) under the following conditions: initial denaturation at 95 °C for 3 min, followed by 27 cycles of denaturing at 95 °C for 30 s, annealing at 55 °C for 30 s and extension at 72 °C for 45 s, and single extension at 72 °C for 10 min, and end at 4 °C. PCR reactions were performed in triplicate 20 µL mixtures. Then, we extracted the PCR product from 2% agarose gel and purified it by using the AxyPrep DNA Gel Extraction Kit (Axygen Biosciences, Union City, CA, USA). Finally, purified amplicons were pooled in equimolar and paired-end sequenced (2 × 300) on an Illumina MiSeq platform (Illumina, San Diego, USA).

### Amplicon sequence processing and analysis

After demultiplexing, we merged the resulting sequences with FLASH (v1.2.11) [20] and quality filtered with fastp (0.19.6) [21]. Then the high-quality sequences were de-noised using DADA2 [22] plugin in the Qiime2 [23] pipeline with recommended parameters, which obtains single nucleotide resolution based on error profiles within samples. DADA2 de-noised sequences are usually called ASVs. To minimize the effects of sequencing depth on alpha and beta diversity measure, the number of sequences from each sample was rarefied to 18750, which still yielded an average Good’s coverage of 99.89%. Taxonomic assignment of ASVs was performed using the Naive bayes consensus taxonomy classifier implemented in Qiime2 and the SILVA 16S rRNA database (v138). α-diversity was measured to estimate the microbial communities’ diversity, including microbial community richness (Ace, Sobs) and diversity (Shannon, Simpson). β-diversity was generated on the basis of bray curtis algorithms and reported according to principal coordinate analysis (PCoA) and partial least squares discriminant analysis (PLS-DA) [24]. We performed a random 70%/30% split for training set and testing set in sparse PLS-DA (sPLS-DA) with the 10 × 5-fold cross-validation via the Mixomics mixMC package [25]. The key bacterial taxa responsible for discrimination between CUMS and CON groups (linear discriminant analysis [LDA] score >2 and *p* value < 0.05) were identified using subsequent linear discriminant analysis effect size (LEfSe) [26]. We also used PICRUSt to predict the microbial functions from the microbial data, and then used LEfSe to identify the significantly different functions between CUMS and CON groups (LDA score >2 and *p* value < 0.05) based on KEGG database (https://www.genome.jp/kegg/).

### Targeted metabolomics profiling

Details of the liquid chromatography–mass spectrometry (LC–MS) analysis have been reported in our previous studies [19, 27]. Briefly, the luminal samples were prepared by homogenization, dissociation, and centrifugation. The LC–MS analysis was conducted using an ultra-high performance liquid chromatography (1290 Infinity LC, Agilent Technologies) and quadrupole time-of-flight (AB SCIEX QQQ 5500). 5500 QTRAP (AB SCIEX) was performed in positive and negative switch mode. Peak chromatographic area and retention time were analyzed with Multiquant software. The standard substance of 34 metabolites involved in carbohydrate or energy metabolism pathways was used to calculate the retention time and identify metabolites (Table S1).

### Statistical analysis

Statistical analyses were done with IBM SPSS Statistics for Windows, Version 25.0 (IBM Corp., Armonk, NY, USA). Wilcoxon Signed Rank Test was conducted to compare the species richness indices (Ace and Sobs), species diversity indices (Shannon and Simpson) and the level of 34 metabolites between CUMS and CON groups, as the distribution of the difference between the two groups’ means cannot be assumed to be normally distributed. Spearman’s correlation was conducted to calculate the relationship of the relative abundances of discriminative ASVs with each other, and also with the depressive-like behaviors by using Benjamini–Hochberg false discovery rate correction procedure to control the multiple testing. Statistical significance level was set at *p* value < 0.05. The investigators were not blinded to the group classification while analyzing the data.

## Results

### Behavioral characteristics of the CUMS monkey model

**Fig. 1:**
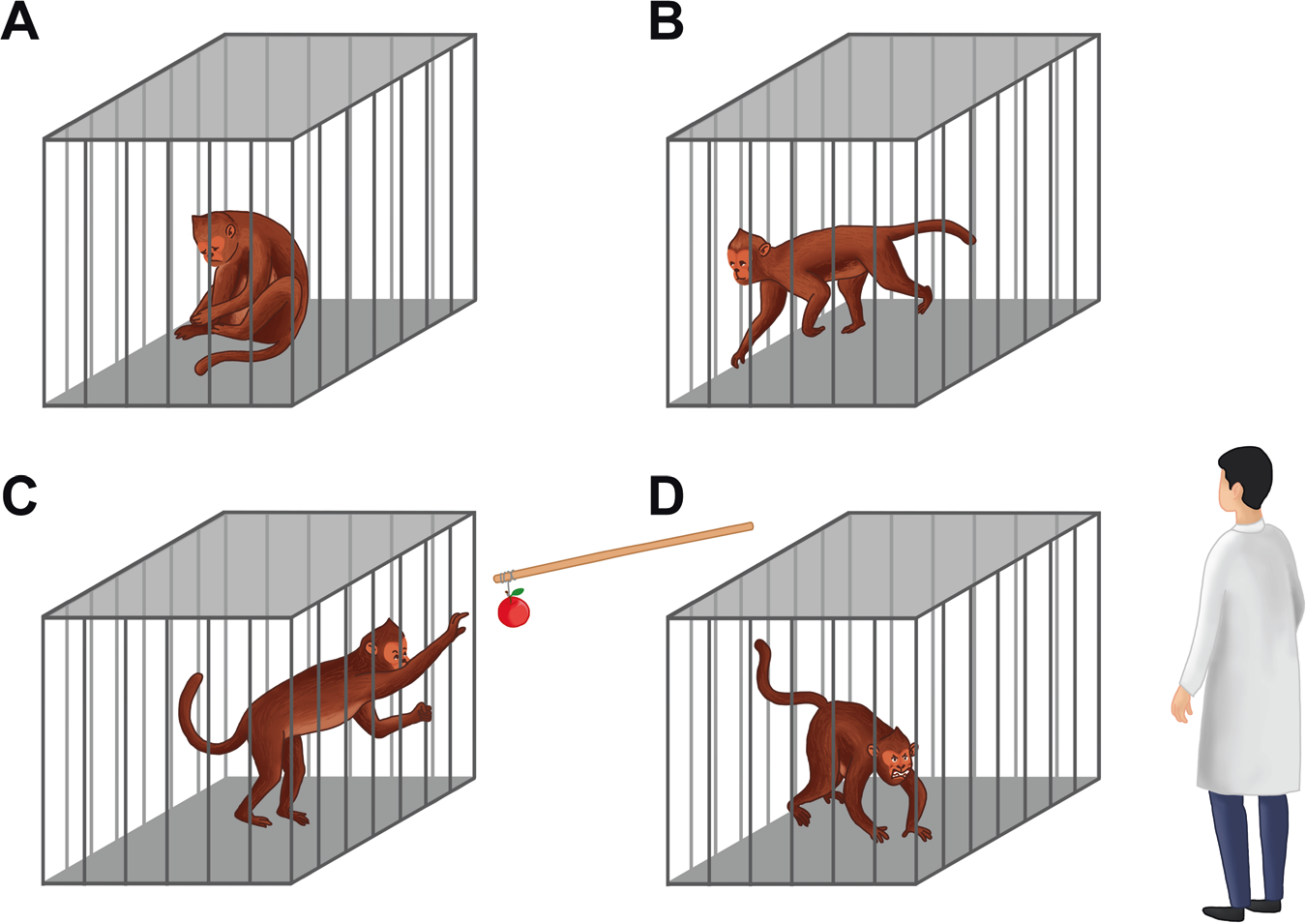
Representative behavior observation or tests of adolescent cynomolgus macaques with depressive-like behaviors. **A** Huddle posture (self-clasping with head at or below the shoulders during the waking state). **B** Locomotion (walking in the cage). **C** Attempt for apple (trying to touch the apple). **D** Fear grimace (a large grin-like facial expression showing the teeth).

Detailed results of behavioral observations and tests were previously reported [19]. Briefly, after exposed to multiple unpredicted mild stressors for five cycles over 55 days, we found that the CUMS animals were characterized by significantly higher frequency and longer duration of huddle posture (Fig. 1A), and lower frequency and shorter duration of locomotion (Fig. 1B). Moreover, CUMS animals showed significantly fewer attempts for the apple in the AAT (Fig. 1C) and significantly more anxiety-like behaviors in the HIT (Fig. 1D). These findings suggested that animals in CUMS group showed depressive-like behaviors.

### Gut microbial composition of CUMS and CON animals

**Fig. 2:**
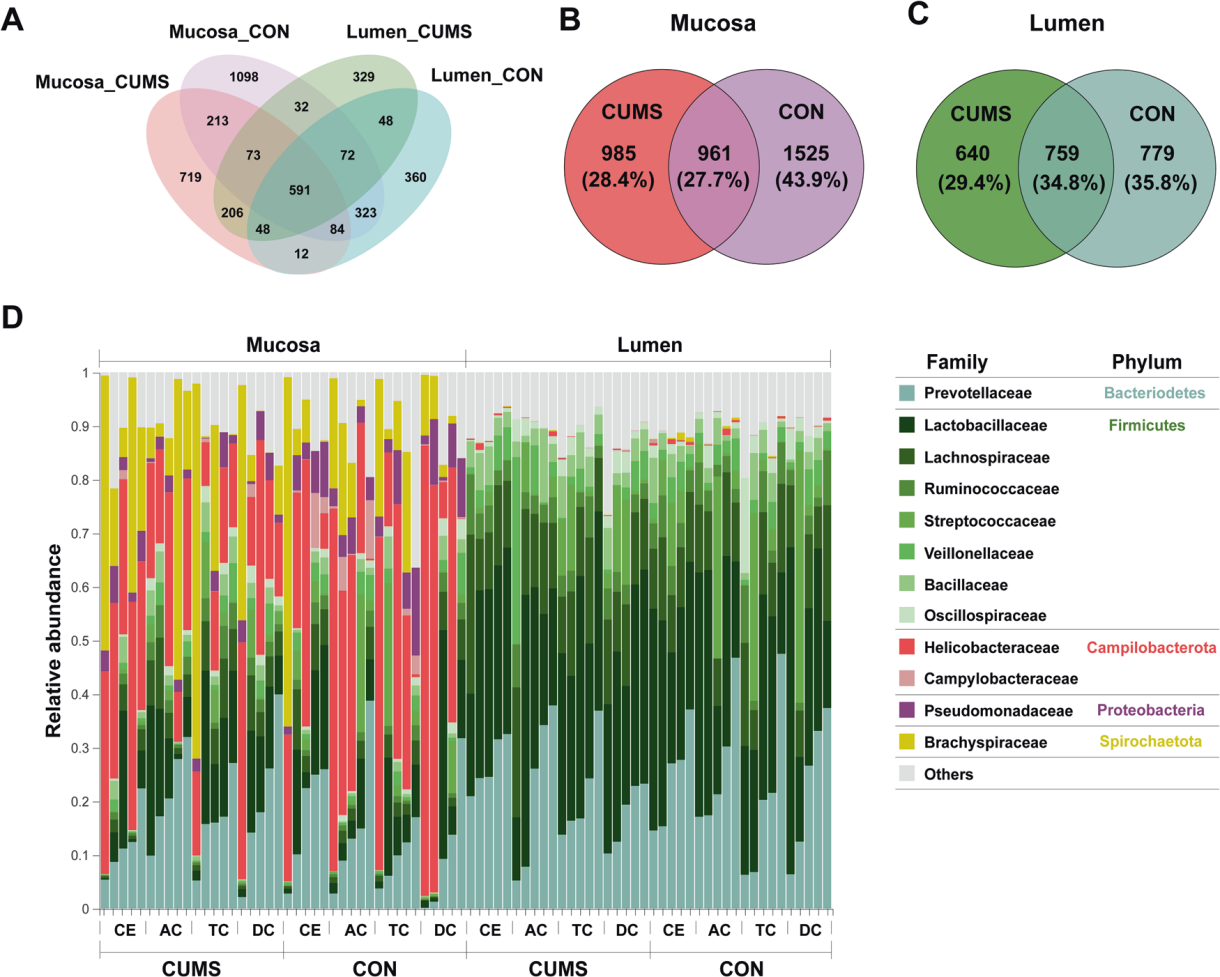
Comparison of the microbial composition between CUMS and CON groups. **A** Venn diagram depicting ASV richness and the overlap in microbial communities in mucosa and lumen between CUMS and CON groups. **B** Venn diagram depicting ASV richness and the overlap in microbial communities in mucosa between CUMS (left) and CON groups (right). **C** Venn diagram depicting ASV richness and the overlap in microbial communities in lumen between CUMS (left) and CON groups (right). **D** Family-level relative abundance of microbial composition in mucosa (left) and of lumen (right) of cecum (CE), ascending colon (AC), transverse colon (TC), and descending colon (DC) in all the ten cynomolgus macaques.

Using 16S rRNA gene sequencing, we identified a total of 2,608,415 high-quality reads across all 80 samples. These reads were clustered into 4208 ASVs, whereas 2030 (48.2%) and 737 (17.5%) ASVs were unique to mucosa and lumen, respectively (Fig. 2A). In the mucosa, 961 (27.7%) ASVs were shared between CUMS and CON groups, while 985 (28.4%) and 1525 (43.9%) ASVs were unique to CUMS group and CON group, respectively (Fig. 2B). In the lumen, 759 (34.8%) ASVs were shared between CUMS and CON groups, while 640 (29.4%) and 779 (35.8%) ASVs were unique to CUMS and CON groups, respectively (Fig. 2C). Here, the gut microbiome was mainly composed of 12 families in 5 phylums (Fig. 2D). At phylum level, Bacteroidetes dominated in both lumen and mucosa, while Firmicutes were more abundant in the lumen, and Campilobacterota and Spirochaetae were more abundant in the mucosa. At family level, Prevotellaceae was dominated in both lumen and mucosa, while Lactobacillaceae and Lachnospiraceae were more abundant in lumen, and Helicobacteraceae and Brachyspiraceae were more abundant in mucosa. Within-sample (α) phylogenetic diversity analysis in four indices (Ace, Shannon, Sobs, and Simpson) showed that there was no difference between two groups in mucosa (Fig. S1A) and lumen (Fig. S1B).

### The alterations of gut microbial composition in CUMS animals

At the ASV-level, PCoA analysis (Fig. S2A) showed that the gut microbial composition of CUMS group was significantly different from CON group in mucosa and lumen (ANOSIM, *p* value = 0.0010, *R* = 0.3202). PLS-DA analysis revealed the microbial composition in mucosa and lumen of CUMS and CON groups clustered separately (Fig. S2B). Based on the balanced error rate (Fig. S3A) in sPLS-DA in training set, two components were sufficient for our final sPLS-DA model to reach optimal performance (Fig. S3B). The receiver operating characteristic (ROC) curve in training set showed that Lumen_CUMS, Lumen_CON, Mucosa_CUMS, and Mucosa_CON were classified from others with the area under curve (AUC) of 0.9932, 0.9041, 0.8061 and 0.8980, respectively (Fig. S3C), as well as in testing set showed that Lumen_CUMS, Lumen_CON, Mucosa_CUMS, and Mucosa_CON were classified from others with the AUC of 0.9444, 0.8000, 0.6389, and 0.9160, respectively (Fig. S3D).

**Fig. 3:**
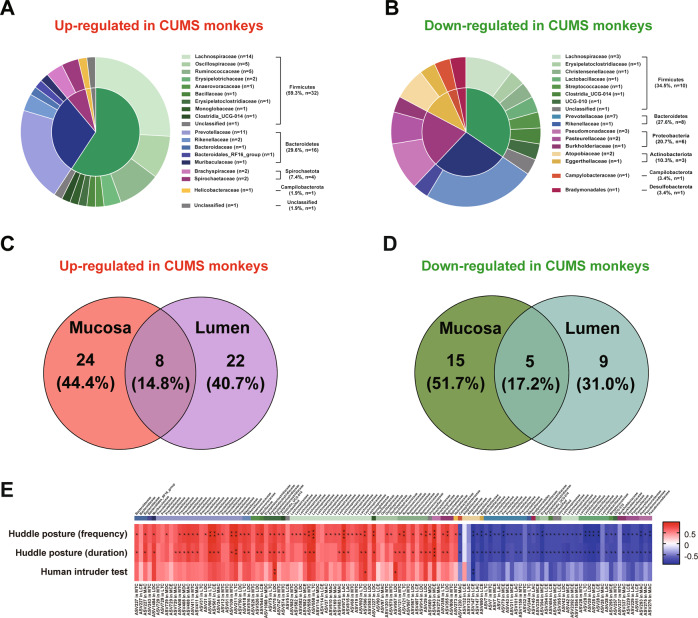
The discriminative ASVs between CUMS and CON groups, and the association between discriminative ASVs and depressive-like behaviors. **A** The pie chart of upregulated ASVs in CUMS macaques at phylum and family level. **B** The pie chart of downregulated ASVs in CUMS macaques at phylum and family level. **C** Venn diagram depicting the overlap in upregulated discriminative ASVs in mucosa and lumen. **D** Venn diagram depicting the overlap in downregulated discriminative ASVs in mucosa and lumen. **E** The association between depressive-like behaviors and discriminative ASVs at mucosa of cecum (MCE), ascending colon (MAC), transverse colon (MTC), and descending colon (MDC), and lumen of cecum (LCE), ascending colon (LAC), transverse colon (LTC), and descending colon (LDC).

To characterize the distinct microbial compositions between CUMS and CON groups in detail, we further identified key discriminative ASVs using LEfSe analysis (LDA >2 and *p* value < 0.05). In total, 83 dynamically changed ASVs in all eight sites (lumen and mucosa of cecum, ascending colon, transverse colon, and descending colon) between CUMS and CON groups (Table S2). The majority of the discriminative ASVs were belonging to Firmicutes (42/83, 50.6%) and Bacteriodetes (24/83, 28.9%) at the phylum level, as well as Prevotellaceae (18/83, 21.7%) and Lachnospiraceae (17/83, 20.5%) at the family level. The CUMS animals were characterized by 54 increased ASVs (Fig. 3A) mainly belonging to Lachnospiraceae (14 ASVs) and Prevotellaceae (11 ASVs), and 29 decreased ASVs (Fig. 3B) mainly belonging to Prevotellaceae (7 ASVs) comparing with CON group. However, only 13 (15.7%) discriminative ASVs were found to be significantly changed in both lumen and mucosa, 8 (14.8%) of which were upregulated (Fig. 3C) in CUMS animals and 5 (17.2%) were downregulated (Fig. 3D), namely those belonging to Firmicutes (10 ASVs) and Bacteriodetes (3 ASVs) (Table S3). Interestingly, ASV28 belonging to family Lactobacillaceae (phylum Firmicutes) was found significantly decreased in seven gastrointestinal sites (Table S2). In addition, we found that the majority (69/83, 83.1%) of discriminative ASVs, mainly belonging to Firmicutes (34/69, 49.3%) and Bacteriodetes (21/69, 30.4%), correlated with the core depressive-like behavioral phenotype (huddle posture) at least one gastrointestinal site (Fig. 3E). At the family level, twelve (12/15, 80.0%) discriminative ASVs belonging to Lachnospiraceae positively correlated with huddle posture. While 10 (10/16, 62.5%) discriminative ASVs belonging to Prevotellaceae positively correlated with huddle posture, and 6 (6/16, 37.5%) discriminative ASVs belonging to Prevotellaceae negatively.

### The co-occurrence network reflecting microbial changes across four gastrointestinal locations in CUMS animals

**Fig. 4:**
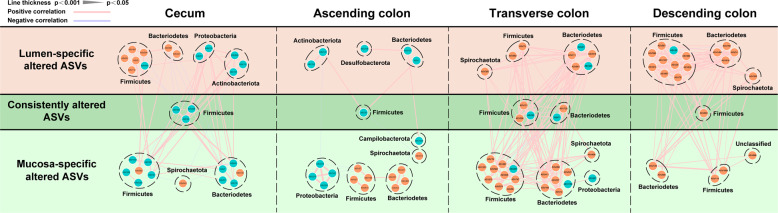
The co-occurrence network reflecting microbial changes between CUMS and CON groups in cecum, ascending colon, transverse colon and descending colon. Red dots represent enriched ASVs in CUMS group relative to CON group; blue dots represent depleted ASVs in CUMS group relative to CON group; ASVs annotated to phylum level were profiled. Edges between dots represent Spearman’s correlation (positive: light red; negative: light blue), edges thickness indicate correlation value (*r*).

Four co-occurrence network analyses provided an explanation of the statistical covariation among discriminative ASVs in cecum, ascending, transverse and descending colons (Fig. 4). Overall, discriminative ASVs constructed relatively complex and diverse covarying networks, and the majority of discriminative ASVs were positively correlated with each other. All the consistently discriminative ASVs in both mucosa and lumen belonged to Firmicutes and Bacteriodetes. Most importantly, Firmicutes and Bacteriodetes generated a characteristic covarying network in cecum and colons. For instance, in lumen of descending colon, nine lumen-specific discriminative ASVs in Firmicutes (ASV29, 75, 526, 567, 593, 596, 914, 957, and 1227) and four ASVs in Bacteriodetes (ASV31, 516, 750, and 1484) were mostly positively covaried with each other, and were also positively covaried with mucosa-specific discriminative ASVs in Firmicutes (ASV114 and 1068) and Bacteriodetes (ASV408 and 729).

### The altered microbiome associated carbohydrate and energy metabolism pathways in CUMS animals

**Fig. 5:**
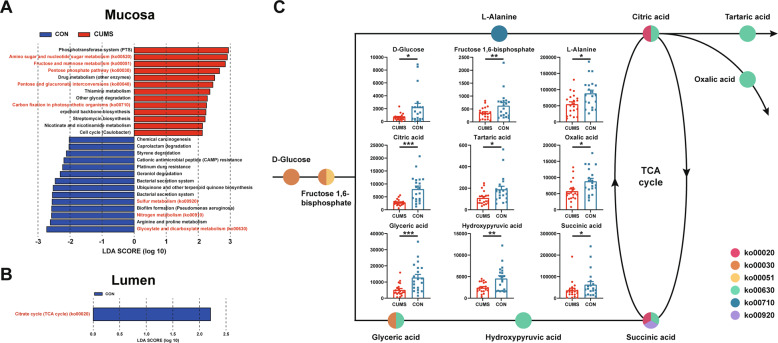
Different altered discriminative ASVs and predicted community functions in lumen and mucosa. **A** The dynamically changed mucosal predicted community functions between CUMS (red) and CON (green) groups. **B** The dynamically changed luminal predicted community functions between CUMS (red) and CON (green) groups. **C** A simplified interaction diagram of nine depleted metabolites involved in carbohydrate and energy metabolism pathways.

Using PICRUSt (LDA >2 and *p* value < 0.05), we found a total of 27 significantly different microbiome community functions between CUMS and CON groups in mucosa, 13 of which were enriched in CUMS group and 14 of which were enriched in CON group (Fig. 5A). However, only Citrate cycle (TCA cycle) was observed to be enriched in CON animals (Fig. 5B). These altered pathways in mucosa mainly involved in 6 carbohydrate and 3 energy metabolism pathways, including amino sugar and nucleotide sugar metabolism (ko00520), fructose and mannose metabolism (ko00051), pentose phosphate pathway (ko00030), pentose and glucuronate interconversions (ko00040), glyoxylate and dicarboxylate metabolism (ko00630), citrate cycle (TCA cycle) (ko00020), carbon fixation in photosynthetic organisms (ko00710), sulfur metabolism (ko00920), and nitrogen metabolism (ko00910). Then, the level of 34 metabolites involved in 6 carbohydrate and 3 energy metabolism pathways were measured in luminal contents by targeted metabolomics profiling (Table S1). A total of 27 metabolites were identified, and 9 of them (D-glucose, fructose 1,6-bisphosphate, L-alanine, citric acid, tartaric acid, oxalic acid, glyceric acid, glyceric acid, hydroxypyruvic acid, and succinic acid) were found to be depleted in CUMS animals (Fig. 5C). The results of the remaining non-significantly altered 18 metabolites were shown in Fig. S4.

## Discussion

**Fig. 6:**
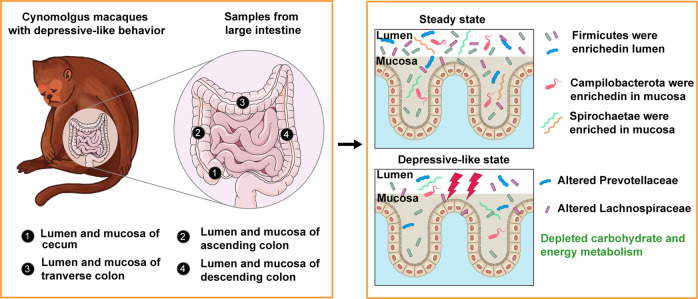
Schematic diagram summarizing the findings of this study. The cynomolgus macaques with depressive-like behaviors were characterized by different alterations in mucosal and luminal covarying ASVs in cecum, ascending, tranverse and descending colon, which were mainly belonging to Prevotellaceae and Lachnospiraceae at the family level. The carbohydrate and energy metabolism were also depleted in cynomolgus macaques with depressive-like behaviors.

Growing evidence suggests that alterations in gut microbiome composition and function may contribute to depression pathology, but few studies have focused on the gut mucosal and luminal microbiome. Here, we observed that CUMS animals with depressive-like behaviors were characterized by different alterations in mucosal and luminal covarying ASVs, which were mainly belonging to Firmicutes and Bacteriodetes at the phylum level, as well as Prevotellaceae and Lachnospiraceae at the family level. Moreover, the alterations in gut microbiome composition were highly correlated with the depressive-like behavioral phenotype (huddle posture). A total of six carbohydrate and three energy metabolism pathways were predicted to be altered in CUMS animals, and nine metabolites involved in these pathways were found to be depleted in CUMS animals, consistent with the PICRUSt analysis. We summarized these findings of this study in a schematic diagram (Fig. 6). To our knowledge, this is the first study to examine the microbial compositions and metabolism functions comprehensively in cynomolgus macaques with depressive-like behavior and healthy controls in multiple gastrointestinal locations of the large intestine.

Fecal microbiome compositional changes have been demonstrated in MDD patients among several previous clinical studies [28]. However, divergent results occurred across studies due to differences in sample recruitment, sequencing and statistical methodology [28]. Overall, at the phylum level, the largest numbers of differentiating taxa were within phylum Firmicutes and Bacteriodetes [29, 30], which can be recovered after antidepressant treatment [31, 32]. At the family level, Lachnospiraceae differentiated between MDD and healthy controls (HCs) with a split in directionality. For example, our group reported Lachnospiraceae was upregulated in MDD patients [8], while Naseribafrouei et al. reported that this family was downregulated [30]. Additionally, MDD was characterized by depleted family Prevotellaceae relative to HCs as well [32, 33]. At the genus level, various genera were reported to be differentiated between MDD and HCs, such as Alistipes and Oscillibacter [28]. Moreover, the genus Coprococcus and Dialister were found to be two microbial markers that distinguish patients with MDD and bipolar disorder in a recent study from our group [34]. Consistent with clinical findings, the microbial composition of macaques after CUMS stress were significantly different from the control animals, which characterized by mucosal and luminal alterations in Firmicutes and Bacteriodetes at the phylum level, and Prevotellaceae and Lachnospiraceae at the family level. In line with our finding, macaques experiencing social stress were also found significant changes in microbial composition, which were mainly assigned to Firmicutes compared with HCs [27]. Interestingly, in this study, ASV28 belonging to genus Lactobacillus was significantly decreased in the whole large intestinal mucosal and luminal sites. A similar result was reported in recent published meta-analysis that the abundance of genera Lactobacillus was lower in MDD patients [35], and was also consistent with rodent stress literature [36]. Moreover, the potential antidepressant effects of the Lactobacillus for MDD patients have been previously documented [37, 38]. Thus, both fecal samples of human, as well as mucosal and luminal samples of NHP have consistently indicated that disturbances of microbial composition in phylum Firmicutes and Bacteriodetes may be a hallmark of depression.

The cooperative status of gut microbes is important in the maintenance of human homeostasis, and the dysbiosis of the microbial ecosystem may lead to the illness of the host [39]. The covariant networks of fecal microbes were previously presented in MDD and bipolar disorder (BD) patients [34]. In MDD, positive correlation networks were mainly found in Operational Taxonomic Units (OTUs) belonging to Bacteroidaceae at the family level, and Bacteriodetes at the phylum level. In BD, the correlation networks were relatively complex and diverse mainly in OTUs belonging to Prevotellaceae at the family level, and also Bacteriodetes at the phylum level. Consistently, the animals with depressive-like behaviors had a positive correlation network among differential covarying ASVs, which were mainly assigned to Firmicutes and Bacteriodetes in cecum and colons comparing with the HCs in this study. These findings suggested that all these discriminative ASVs may play a cooperative role in the large intestine microbial environment of depression, and disturbed Firmicutes and Bacteriodetes ASVs were a hallmark in the gut ecosystem of depression. The underlying pathogenesis of depression in the gut microbiota may result from a wider modification of the microbial ecosystem rather than just a single gut microbe.

It has been demonstrated that the metabolic pathways in the host can be significantly shaped by mucosal and luminal bacteria [12]. The functions of mucosal and luminal microbes has been observed across the intestinal tract in healthy mammals such as human [40] and macaques [12]. The microbial compositions of mucosa and lumen have a profoundly beneficial role in energy [41] and immunity [42], and also in maintaining homeostasis between intestinal epithelial cells and microbes [43]. Recently, emerging evidence suggests that the mucosal-associated microbiome is relevant to the pathogenesis of gastrointestinal diseases such as Crohn’s disease [11], cirrhosis [44] and colorectal cancer [45], and also relevant to anxiety [46] and stroke [47] based on rodent depression model. Furthermore, these signatures of gut microbiome compositional alterations in the mucosa may be site specific and not detected in stool samples [11]. Similar to previous studies, this study revealed that the predicted community functions of the mucosal- and luminal-associated microbiome play an essential role in the pathology of depressive-like behaviors. In detail, altered carbohydrate and energy metabolism were predicted in mucosa of large intestine, and only Citrate cycle (TCA cycle), which was also belonging to carbohydrate metabolism, was also predicted to be altered in the lumen of large intestine, and nine depleted metabolites involved in these carbohydrate and energy pathways were verified in CUMS animals. These findings could be explained by the disturbance of the Firmicutes and Bacteriodetes, which have emerged as key regulators of host carbohydrate and energy metabolism [31, 48]. The plasma metabolites of carbohydrate metabolism were found to be correlated with the altered gut bacteria in irritable bowel syndrome patients with comorbid depression [49]. In addition, adenosine triphosphate (ATP), which is pivotal in carbohydrate metabolism, has been confirmed as a factor involved in the biological mechanisms of MDD [50]. As early as in 1984, significantly altered energy metabolism was identified in the cerebrospinal fluid and blood of MDD patients [51], as well in more recent rodent [52] and NHP depression models [53]. Furthermore, the energy metabolism and microbiome were consistently changed in rats with depressive-like behaviors after chronic paradoxical sleep deprivation [54]. Here, we found that CUMS macaques with depressive-like behaviors were characterized by the alteration of mucosal carbohydrate and energy metabolism, which may provide a new insight into pathogenesis of MDD.

There are some potential limitations in this study. First, the sample size of cynomolgus macaques was relatively small in this study; and this was mitigated by the use of a paired design. Moreover, our results were consistent with the significant results of depressive-like behavior that were found among CUMS and CON macaques in a previous study [19]. We suggest that further longitudinal studies with larger samples should be performed to validate our findings by dividing the data into training and testing data with larger samples. Second, to minimize the disturbance by menstrual cycles [55], only male animals were included in this study: further studies should be conducted to identify whether these observations will generalize to females. Third, the targeted metabolomics profiling was not applied in mucosal samples as the results of metabolomics profiling would be inevitably altered by the hosts’ intestinal epithelial cells. Further shotgun metagenomics studies should be performed to avoid the influence of the host. Fourth, in this study, gut microbiota and metabolism in cynomolgus macaques may be affected by fasting or water deprivation. However, these stressors are the most common and important components in the CUMS protocol [56]. Thus, we reduced the density of these stressors to minimize the potential effects. Last but not least, small intestine samples were not collected because it is difficult to obtain luminal samples of small intestine after fasting and before euthanasia, and this study mainly focused on the difference between mucosal and luminal microbiome in depression.

In summary, by using a validated non-human primate depression model, we found that CUMS macaques with depressive-like behaviors showed different alterations in mucosal and luminal microbial composition of covarying ASVs in Firmicutes and Bacteriodetes at the phylum level, and in Prevotellaceae and Lachnospiraceae at the family level. Moreover, the majority of the predicted alterations in microbial community functions were involved in carbohydrate and energy metabolism pathways, and nine metabolites involved in these pathways were verified to be depleted in CUMS animals. These findings advance our understanding of how the mucosal and luminal microbiome may contribute to the pathophysiology of depression by identifying pathways leading to alterations in metabolism in a depressive-like state.

## Supplementary information

Supplementary legends

Table S1

Table S2

Table S3

Figure S1

Figure S2

Figure S3

Figure S4
