# Supplementary material for: Biogeography of the large intestinal mucosal and luminal microbiome in cynomolgus macaques with depressive-like behavior
Source: Mol Psychiatry. 2021 Nov 1;27(2):1059–67. doi: 10.1038/s41380-021-01366-w (PMC9054659; doi:10.1038/s41380-021-01366-w)
Supplement: Supplementary file 1 — Supplementary legends [file 41380_2021_1366_MOESM1_ESM.docx]

**Figure S1.** α-diversity analysis of microbial community richness (Chao, Ace) and diversity (Shannon, Simpson) between CUMS and CON groups in mucosa (A) and lumen (B).

**Figure S2.** Principal coordinate analysis (A) and partial least squares discriminant analysis (B) for the mucosal and luminal microbial signatures between CUMS and CON groups.

**Figure S3.** (A) Estimated classification balanced error rates in training set. (B) Sparse partial least squares discriminant analysis plot based on two components in training set. (C) The receiver operating characteristic curve in training set including two components from the final model. (D) The receiver operating characteristic curve in testing set including two components from the final model. PPV: positive predictive value; NPV: negative predictive value.

**Figure S4.** The results of the non-significantly altered 18 metabolites involved in carbohydrate and energy metabolism pathways.

**Table S1.** The detail of standard substance of 34 metabolites used in targeted metabolism profiling.

**Table S2.** The discriminative ASVs in mucosa and lumen of cecum, ascending colon, transverse colon, and descending colon identified by LEfSe.

**Table S3.** The list for the lumen-specific, mucosa-specific, and consistently altered ASVs.
