# Supplementary material for: Biogeography of the large intestinal mucosal and luminal microbiome in cynomolgus macaques with depressive-like behavior
Source: Mol Psychiatry. 2021 Nov 1;27(2):1059–67. doi: 10.1038/s41380-021-01366-w (PMC9054659; doi:10.1038/s41380-021-01366-w)
Supplement: Supplementary file 2 — Table S1 [file 41380_2021_1366_MOESM2_ESM.docx]

**Table S1.** The detail of standard substance of 34 metabolites used in targeted metabolism profiling.

| **Name** | **KEGG ID** | **Article number (specification)** | **CAS number** | **Brand** | **Metabolism pathway** |
| --- | --- | --- | --- | --- | --- |
| L-Alanine | C00041 | YJ-140624 (100mg) | 56-41-7 | NIFDC | ko00710 |
| Hydroxypyruvic acid | C00168 | 06372-5MG-F | 1113-60-6 | sigma | ko00630 |
| D-Glucose | C00221 | ZDR-C14027000 (250mg) | 50-99-7 | DR,E | ko00030 |
| Fructose 1,6-bisphosphate | C05378 | F111301 (1000mg) | - | Aladdin | ko00051, ko00030 |
| Glyceric acid | C00258 | HY-W018035 (1000mg) | 473-81-4 | MCE | ko00030, ko00630 |
| Tartaric acid | C00898 | CCHM700638 (1000mg) | 87-69-4 | CAS testing | ko00630 |
| Citric acid | C00158 | 791725 (500g) | 77-92-9 | Sigma | ko00630, ko00020 |
| Oxalic acid | C00209 | ZDR-C15775000 (250mg) | 144-62-7 | DR,E | ko00630 |
| Succinic acid | C00042 | 14078 (1000mg) | 110-15-6 | Fluka | ko00630, ko00920, ko00020 |
| Glycine | C00037 | YJ-140624 (100mg) | 56-40-6 | NIFDC | ko00630 |
| L-Glutamic acid | C00025 | YJ-140624 (100mg) | 56-86-0 | NIFDC | ko00630, ko00910 |
| L-Glutamine | C00064 | YJ-140624 (100mg) | 56-85-9 | NIFDC | ko00630, ko00910 |
| L-Aspartic acid | C00049 | A9256 (100g) | 56-84-8 | sigma | ko00710 |
| L-Serine | C00065 | YJ-140624 (100mg) | 56-45-1 | NIFDC | ko00630 |
| N-Acetyl-D-glucosamine | C00140 | HY-A0132 (500mg) | 7512-17-6 | MCE | ko00520 |
| D-Fructose | C02336 | ZT-71220 (100mg) | 57-48-7 | zzstandard | ko00520 |
| D-Gluconolactone | C00198 | HY-I0301 (500mg) | 90-80-2 | MCE | ko00030 |
| D-Xylose | C00181 | ZDR-C17946000 (250mg) | 58-86-6 | DR,E | ko00520, ko00040 |
| Rhamnose | C00507 | ZT-20711 (20mg) | 10030-85-0 | zzstandard | ko00051 |
| cis-Aconitic acid | C00417 | A3412 (1000mg) | 585-84-2 | Sigma | ko00630, ko00020 |
| Fumaric acid | C00122 | F1506 (25g) | 110-17-8 | Sigma | ko00020 |
| Isocitric acid | C00311 | I1252 (25g) | 320-77-4 | Sigma | ko00630, ko00020 |
| L-Malic acid | C00149 | 112577 (25g) | 97-67-6 | Sigma | ko00630, ko00710, ko00020 |
| Pyruvic acid | C00022 | 107360 (25g) | 127-17-3 | Sigma | ko00030, ko00040, ko00630, ko00710, ko00020 |
| α-Ketoglutaric acid | C00026 | 75890 (25g) | - | Sigma | ko00040, ko00630, ko00020 |
| Thiamine pyrophosphate | C00068 | C8754 (5000mg) | 136-09-4 | Sigma | ko00020 |
| Oxaloacetic acid | C00036 | O4126 (1000g) | 328-42-7 | Sigma | ko00630, ko00710, ko00020 |
| Dihydroxyacetone phosphate | C00111 | 37442 (100mg) | 57-04-5 | Sigma | ko00051, ko00040, ko00710 |
| Acetyl-CoA | C00024 | A2056 (25mg) | 72-89-9 | Sigma | ko00630, ko00020 |
| Succinyl-CoA | C00091 | S1129 (25mg) | 604-98-8 | Sigma | ko00630, ko00020 |
| Indoxyl-β-D-glucuronide | C03033 | 373680 (250mg) | 35804-66-1 | J&K | ko00040 |
| L-Homoserine | C00263 | H6515 (10mg) | 672-15-1 | sigma | ko00920 |
| L-Cysteine | C00097 | C1276 (10g) | 52-90-4 | Sigma | ko00920 |
| Taurine | C00245 | T0625 (10g) | 107-35-7 | sigma | ko00920 |
