# Supplementary material for: Biogeography of the large intestinal mucosal and luminal microbiome in cynomolgus macaques with depressive-like behavior
Source: Mol Psychiatry. 2021 Nov 1;27(2):1059–67. doi: 10.1038/s41380-021-01366-w (PMC9054659; doi:10.1038/s41380-021-01366-w)
Supplement: Supplementary file 3 — Table S2 [file 41380_2021_1366_MOESM3_ESM.docx]

**Table S2.** The discriminative ASVs in mucosa and lumen of cecum, ascending colon, transverse colon, and descending colon identified by LEfSe.

| **Number of changed sites** | **ASV** | **Phylum** | **Family** | **Mucosa of cecum**  **[LDA (P-value)]** | **Lumen of cecum [LDA (P-value)]** | **Mucosa of ascending colon [LDA (P-value)]** | **Lumen of ascending colon [LDA (P-value)]** | **Mucosa of transverse colon [LDA (P-value)]** | **Lumen of transverse colon [LDA (P-value)]** | **Mucosa of descending colon [LDA (P-value)]** | **Lumen of descending colon [LDA (P-value)]** |
| --- | --- | --- | --- | --- | --- | --- | --- | --- | --- | --- | --- |
| 7 | **ASV28** | Firmicutes | Lactobacillaceae | 3.6384  (0.0150) | 3.8938  (0.0082) | 2.9982  (0.0343) | 3.4237  (0.0150) | 2.8636  (0.0132) | 3.3166  (0.0082) |  | 3.4057  (0.0071) |
| 5 | **ASV729** | Bacteriodetes | Prevotellaceae | 2.6751  (0.0343) |  | 2.9553  (0.0236) |  | 2.8352  (0.0343) | 2.7912  (0.0186) | 2.9961  (0.0186) |  |
| 4 | **ASV7** | Bacteriodetes | Prevotellaceae | 3.2597  (0.0186) |  |  | 3.5709  (0.0186) | 3.1980  (0.0132) | 3.7787  (0.0186) |  |  |
|  | **ASV582** | Firmicutes | Lachnospiraceae | 3.0036  (0.0186) |  |  |  | 2.7374  (0.0343) |  | 2.5914  (0.0343) | 2.7214  (0.0343) |
| 3 | **ASV408** | Bacteriodetes | Prevotellaceae |  |  | 2.8931  (0.0343) |  | 2.6742  (0.0053) |  | 2.5748  (0.0343) |  |
|  | **ASV227** | Bacteriodetes | Bacteroidaceae |  | 2.8516  (0.0445) | 3.1939  (0.0082) |  | 2.6473  (0.0088) |  |  |  |
| 2 | **ASV121** | Firmicutes | Ruminococcaceae |  |  |  |  | 2.9329  (0.0236) | 2.8194  (0.0264) |  |  |
|  | **ASV558** | Firmicutes | Lachnospiraceae |  |  |  |  | 2.9169  (0.0186) | 2.8781  (0.0053) |  |  |
|  | **ASV567** | Firmicutes | Ruminococcaceae |  |  |  |  | 2.7209  (0.0406) |  |  | 2.9125  (0.0236) |
|  | **ASV526** | Firmicutes | Anaerovoracaceae |  |  |  |  | 2.4259  (0.0343) |  |  | 2.6195  (0.0053) |
|  | **ASV337** | Proteobacteria | Burkholderiaceae |  |  | 3.0638  (0.0264) |  | 2.3212  (0.0465) |  |  |  |
|  | **ASV411** | Bacteriodetes | Prevotellaceae |  |  | 3.1589  (0.0236) |  | 3.1622  (0.0264) |  |  |  |
|  | **ASV31** | Bacteriodetes | Prevotellaceae |  |  |  |  |  | 3.3926  (0.0472) |  | 3.6021  (0.0163) |
|  | **ASV75** | Firmicutes | Erysipelotrichaceae |  |  |  |  |  | 3.1117  (0.0090) |  | 3.1342  (0.0090) |
|  | **ASV355** | Spirochaetota | Spirochaetaceae |  |  |  |  |  | 2.6985  (0.0445) |  | 2.7796  (0.0160) |
|  | **ASV561** | Bacteriodetes | Prevotellaceae |  | 2.8560  (0.0186) |  |  |  | 2.7415  (0.0186) |  |  |
|  | **ASV114** | Firmicutes | Lachnospiraceae |  | 3.5795  (0.0472) |  |  |  |  | 2.7974  (0.0278) |  |
|  | **ASV42** | Firmicutes | Lachnospiraceae | 2.6041  (0.0278) |  |  |  |  |  |  | 3.0870  (0.0472) |
|  | **ASV395** | Firmicutes | f__unclassified_o__Lactobacillales | 2.5868  (0.0186) | 2.8243  (0.0186) |  |  |  |  |  |  |
|  | **ASV394** | Firmicutes | Erysipelatoclostridiaceae | 2.8997  (0.0053) | 2.6580  (0.0186) |  |  |  |  |  |  |
|  | **ASV132** | Actinobacteriota | Atopobiaceae |  | 2.9447  (0.0088) |  | 2.6317  (0.0445) |  |  |  |  |
|  | **ASV229** | Proteobacteria | Pasteurellaceae |  | 3.0203  (0.0150) |  | 2.7192  (0.0186) |  |  |  |  |
|  | **ASV147** | Actinobacteriota | Atopobiaceae |  | 2.9833  (0.0090) |  | 2.9036  (0.0283) |  |  |  |  |
| 1 | **ASV79** | Bacteriodetes | Prevotellaceae | 2.8528  (0.0053) |  |  |  |  |  |  |  |
|  | **ASV643** | Spirochaetota | Brachyspiraceae | 4.4742  (0.0053) |  |  |  |  |  |  |  |
|  | **ASV43** | Bacteriodetes | Prevotellaceae | 3.1688  (0.0343) |  |  |  |  |  |  |  |
|  | **ASV311** | Bacteriodetes | Prevotellaceae | 2.4814  (0.0343) |  |  |  |  |  |  |  |
|  | **ASV295** | Firmicutes | Streptococcaceae | 2.7516  (0.0343) |  |  |  |  |  |  |  |
|  | **ASV2392** | Firmicutes | Lachnospiraceae | 2.4695  (0.0186) |  |  |  |  |  |  |  |
|  | **ASV1054** | Firmicutes | Lachnospiraceae | 2.8269  (0.0186) |  |  |  |  |  |  |  |
|  | **ASV338** | Firmicutes | Christensenellaceae | 2.4078  (0.0264) |  |  |  |  |  |  |  |
|  | **ASV90** | Firmicutes | Oscillospiraceae |  | 2.9161  (0.0343) |  |  |  |  |  |  |
|  | **ASV454** | Firmicutes | f__norank_o__Clostridia_UCG-014 |  | 2.8345  (0.0186) |  |  |  |  |  |  |
|  | **ASV544** | Firmicutes | Bacillaceae |  | 3.1645  (0.0132) |  |  |  |  |  |  |
|  | **ASV51** | Proteobacteria | Pasteurellaceae |  | 3.3040  (0.0186) |  |  |  |  |  |  |
|  | **ASV690** | Actinobacteriota | Eggerthellaceae |  | 2.9165  (0.0053) |  |  |  |  |  |  |
|  | **ASV497** | Firmicutes | Ruminococcaceae |  | 2.8956  (0.0343) |  |  |  |  |  |  |
|  | **ASV5** | Firmicutes | f__unclassified_c__Bacilli |  | 3.8384  (0.0283) |  |  |  |  |  |  |
|  | **ASV34** | Bacteriodetes | Prevotellaceae |  |  | 3.5254  (0.0472) |  |  |  |  |  |
|  | **ASV272** | Spirochaetota | Brachyspiraceae |  |  | 4.2251  (0.0264) |  |  |  |  |  |
|  | **ASV293** | Proteobacteria | Pseudomonadaceae |  |  | 3.1544  (0.0088) |  |  |  |  |  |
|  | **ASV37** | Firmicutes | Lachnospiraceae |  |  | 3.1635  (0.0283) |  |  |  |  |  |
|  | **ASV275** | Proteobacteria | Pseudomonadaceae |  |  | 3.8253  (0.0283) |  |  |  |  |  |
|  | **ASV276** | Proteobacteria | Pseudomonadaceae |  |  | 3.5387  (0.0090) |  |  |  |  |  |
|  | **ASV610** | Firmicutes | Lachnospiraceae |  |  | 3.2141  (0.0186) |  |  |  |  |  |
|  | **ASV169** | Firmicutes | Lachnospiraceae |  |  | 3.0142  (0.0343) |  |  |  |  |  |
|  | **ASV1230** | Campilobacterota | Campylobacteraceae |  |  | 4.0078  (0.0186) |  |  |  |  |  |
|  | **ASV603** | Firmicutes | Lachnospiraceae |  |  | 3.0981  (0.0236) |  |  |  |  |  |
|  | **ASV97** | Firmicutes | Oscillospiraceae |  |  | 2.9774  (0.0472) |  |  |  |  |  |
|  | **ASV972** | Firmicutes | Lachnospiraceae |  |  |  | 2.8873  (0.0186) |  |  |  |  |
|  | **ASV145** | Desulfobacterota | f__norank_o__Bradymonadales |  |  |  | 2.6866  (0.0343) |  |  |  |  |
|  | **ASV815** | Firmicutes | Lachnospiraceae |  |  |  | 2.7496  (0.0186) |  |  |  |  |
|  | **ASV101** | Bacteriodetes | Prevotellaceae |  |  |  | 2.8434  (0.0186) |  |  |  |  |
|  | **ASV747** | Firmicutes | Erysipelotrichaceae |  |  |  |  | 2.5081  (0.0186) |  |  |  |
|  | **ASV257** | Firmicutes | UCG-010 |  |  |  |  | 2.6989  (0.0186) |  |  |  |
|  | **ASV806** | Spirochaetota | Spirochaetaceae |  |  |  |  | 2.4144  (0.0186) |  |  |  |
|  | **ASV574** | Firmicutes | f__norank_o__Clostridia_UCG-014 |  |  |  |  | 2.6541  (0.0186) |  |  |  |
|  | **ASV68** | Firmicutes | Lachnospiraceae |  |  |  |  | 2.6454  (0.0264) |  |  |  |
|  | **ASV243** | Bacteriodetes | Bacteroidales_RF16_group |  |  |  |  | 2.6289  (0.0186) |  |  |  |
|  | **ASV1159** | Bacteriodetes | Prevotellaceae |  |  |  |  | 2.9772  (0.0186) |  |  |  |
|  | **ASV12** | Bacteriodetes | Prevotellaceae |  |  |  |  | 3.9359  (0.0472) |  |  |  |
|  | **ASV1201** | Firmicutes | Oscillospiraceae |  |  |  |  | 2.2427  (0.0343) |  |  |  |
|  | **ASV570** | Firmicutes | Oscillospiraceae |  |  |  |  | 2.6787  (0.0343) |  |  |  |
|  | **ASV61** | Bacteriodetes | Prevotellaceae |  |  |  |  | 2.3786  (0.0186) |  |  |  |
|  | **ASV273** | Campilobacterota | Helicobacteraceae |  |  |  |  | 4.1634  (0.0465) |  |  |  |
|  | **ASV19** | Firmicutes | Lachnospiraceae |  |  |  |  | 2.9697  (0.0472) |  |  |  |
|  | **ASV109** | Bacteriodetes | Prevotellaceae |  |  |  |  | 3.1654  (0.0053) |  |  |  |
|  | **ASV32** | Bacteriodetes | Prevotellaceae |  |  |  |  | 2.7014  (0.0472) |  |  |  |
|  | **ASV244** | Bacteriodetes | Rikenellaceae |  |  |  |  |  | 2.8072  (0.0186) |  |  |
|  | **ASV211** | Bacteriodetes | Prevotellaceae |  |  |  |  |  | 2.8196  (0.0082) |  |  |
|  | **ASV618** | Bacteriodetes | Rikenellaceae |  |  |  |  |  | 3.1659  (0.0053) |  |  |
|  | **ASV592** | Firmicutes | Lachnospiraceae |  |  |  |  |  | 2.8939  (0.0343) |  |  |
|  | **ASV669** | p__unclassified_k__norank_d__Bacteria | f__unclassified_k__norank_d__Bacteria |  |  |  |  |  |  | 2.5836  (0.0186) |  |
|  | **ASV1068** | Firmicutes | Erysipelatoclostridiaceae |  |  |  |  |  |  | 2.6438  (0.0343) |  |
|  | **ASV914** | Firmicutes | Ruminococcaceae |  |  |  |  |  |  |  | 2.7402  (0.0071) |
|  | **ASV596** | Firmicutes | Lachnospiraceae |  |  |  |  |  |  |  | 2.6545  (0.0186) |
|  | **ASV516** | Bacteriodetes | Rikenellaceae |  |  |  |  |  |  |  | 2.7621  (0.0278) |
|  | **ASV957** | Firmicutes | Oscillospiraceae |  |  |  |  |  |  |  | 2.7600  (0.0264) |
|  | **ASV1227** | Firmicutes | Monoglobaceae |  |  |  |  |  |  |  | 2.5112  (0.0186) |
|  | **ASV593** | Firmicutes | Lachnospiraceae |  |  |  |  |  |  |  | 2.5198  (0.0186) |
|  | **ASV750** | Bacteriodetes | Prevotellaceae |  |  |  |  |  |  |  | 2.6508  (0.0343) |
|  | **ASV29** | Firmicutes | Ruminococcaceae |  |  |  |  |  |  |  | 3.1076  (0.0132) |
|  | **ASV1484** | Bacteriodetes | Muribaculaceae |  |  |  |  |  |  |  | 2.4949  (0.0186) |

Red: increased in CUMS group; Green: decreased in CUMS group;
