# Supplementary material for: Biogeography of the large intestinal mucosal and luminal microbiome in cynomolgus macaques with depressive-like behavior
Source: Mol Psychiatry. 2021 Nov 1;27(2):1059–67. doi: 10.1038/s41380-021-01366-w (PMC9054659; doi:10.1038/s41380-021-01366-w)
Supplement: Supplementary file 4 — Table S3 [file 41380_2021_1366_MOESM4_ESM.docx]

**Table S3.** The list for the lumen-specific, mucosa-specific, and consistently altered ASVs.

| **Up-regulated in CUMS monkeys** | | | |
| --- | --- | --- | --- |
|  | **ASV** | **Phylum** | **Family** |
| Consistently altered ASVs | ASV114 | Firmicutes | Lachnospiraceae |
|  | ASV567 | Firmicutes | Ruminococcaceae |
|  | ASV526 | Firmicutes | Anaerovoracaceae |
|  | ASV582 | Firmicutes | Lachnospiraceae |
|  | ASV121 | Firmicutes | Ruminococcaceae |
|  | ASV558 | Firmicutes | Lachnospiraceae |
|  | ASV227 | Bacteriodetes | Bacteroidaceae |
|  | ASV729 | Bacteriodetes | Prevotellaceae |
| Mucosa-specific ASVs | ASV273 | Campilobacterota | Helicobacteraceae |
|  | ASV34 | Bacteriodetes | Prevotellaceae |
|  | ASV747 | Firmicutes | Erysipelotrichaceae |
|  | ASV19 | Firmicutes | Lachnospiraceae |
|  | ASV610 | Firmicutes | Lachnospiraceae |
|  | ASV109 | Bacteriodetes | Prevotellaceae |
|  | ASV643 | Spirochaetota | Brachyspiraceae |
|  | ASV669 | unclassified | unclassified |
|  | ASV806 | Spirochaetota | Spirochaetaceae |
|  | ASV272 | Spirochaetota | Brachyspiraceae |
|  | ASV408 | Bacteriodetes | Prevotellaceae |
|  | ASV37 | Firmicutes | Lachnospiraceae |
|  | ASV574 | Firmicutes | norank_o__Clostridia_UCG-014 |
|  | ASV169 | Firmicutes | Lachnospiraceae |
|  | ASV68 | Firmicutes | Lachnospiraceae |
|  | ASV243 | Bacteriodetes | Bacteroidales_RF16_group |
|  | ASV12 | Bacteriodetes | Prevotellaceae |
|  | ASV1068 | Firmicutes | Erysipelatoclostridiaceae |
|  | ASV1201 | Firmicutes | Oscillospiraceae |
|  | ASV603 | Firmicutes | Lachnospiraceae |
|  | ASV570 | Firmicutes | Oscillospiraceae |
|  | ASV97 | Firmicutes | Oscillospiraceae |
|  | ASV61 | Bacteriodetes | Prevotellaceae |
|  | ASV411 | Bacteriodetes | Prevotellaceae |
| Lumen-specific ASVs | ASV972 | Firmicutes | Lachnospiraceae |
|  | ASV497 | Firmicutes | Ruminococcaceae |
|  | ASV914 | Firmicutes | Ruminococcaceae |
|  | ASV5 | Firmicutes | unclassified |
|  | ASV596 | Firmicutes | Lachnospiraceae |
|  | ASV90 | Firmicutes | Oscillospiraceae |
|  | ASV593 | Firmicutes | Lachnospiraceae |
|  | ASV750 | Bacteriodetes | Prevotellaceae |
|  | ASV516 | Bacteriodetes | Rikenellaceae |
|  | ASV29 | Firmicutes | Ruminococcaceae |
|  | ASV957 | Firmicutes | Oscillospiraceae |
|  | ASV1484 | Bacteriodetes | Muribaculaceae |
|  | ASV31 | Bacteriodetes | Prevotellaceae |
|  | ASV544 | Firmicutes | Bacillaceae |
|  | ASV211 | Bacteriodetes | Prevotellaceae |
|  | ASV561 | Bacteriodetes | Prevotellaceae |
|  | ASV815 | Firmicutes | Lachnospiraceae |
|  | ASV75 | Firmicutes | Erysipelotrichaceae |
|  | ASV1227 | Firmicutes | Monoglobaceae |
|  | ASV592 | Firmicutes | Lachnospiraceae |
|  | ASV355 | Spirochaetota | Spirochaetaceae |
|  | ASV618 | Bacteriodetes | Rikenellaceae |
| **Down-regulated in CUMS monkeys** | | | |
|  | **ASV** | **Phylum** | **Family** |
| Consistently altered ASVs | ASV7 | Bacteriodetes | Prevotellaceae |
|  | ASV28 | Firmicutes | Lactobacillaceae |
|  | ASV42 | Firmicutes | Lachnospiraceae |
|  | ASV395 | Firmicutes | unclassified_o__Lactobacillales |
|  | ASV394 | Firmicutes | Erysipelatoclostridiaceae |
| Mucosa-specific ASVs | ASV295 | Firmicutes | Streptococcaceae |
|  | ASV1159 | Bacteriodetes | Prevotellaceae |
|  | ASV79 | Bacteriodetes | Prevotellaceae |
|  | ASV1230 | Campilobacterota | Campylobacteraceae |
|  | ASV337 | Proteobacteria | Burkholderiaceae |
|  | ASV257 | Firmicutes | UCG-010 |
|  | ASV311 | Bacteriodetes | Prevotellaceae |
|  | ASV32 | Bacteriodetes | Prevotellaceae |
|  | ASV43 | Bacteriodetes | Prevotellaceae |
|  | ASV338 | Firmicutes | Christensenellaceae |
|  | ASV293 | Proteobacteria | Pseudomonadaceae |
|  | ASV275 | Proteobacteria | Pseudomonadaceae |
|  | ASV2392 | Firmicutes | Lachnospiraceae |
|  | ASV276 | Proteobacteria | Pseudomonadaceae |
|  | ASV1054 | Firmicutes | Lachnospiraceae |
| Lumen-specific ASVs | ASV147 | Actinobacteriota | Atopobiaceae |
|  | ASV690 | Actinobacteriota | Eggerthellaceae |
|  | ASV229 | Proteobacteria | Pasteurellaceae |
|  | ASV101 | Bacteriodetes | Prevotellaceae |
|  | ASV244 | Bacteriodetes | Rikenellaceae |
|  | ASV145 | Desulfobacterota | norank_o__Bradymonadales |
|  | ASV51 | Proteobacteria | Pasteurellaceae |
|  | ASV132 | Actinobacteriota | Atopobiaceae |
|  | ASV454 | Firmicutes | norank_o__Clostridia_UCG-014 |
