# Supplementary figures and images for: Biogeography of the large intestinal mucosal and luminal microbiome in cynomolgus macaques with depressive-like behavior
Source: Mol Psychiatry. 2021 Nov 1;27(2):1059–67. doi: 10.1038/s41380-021-01366-w (PMC9054659; doi:10.1038/s41380-021-01366-w)

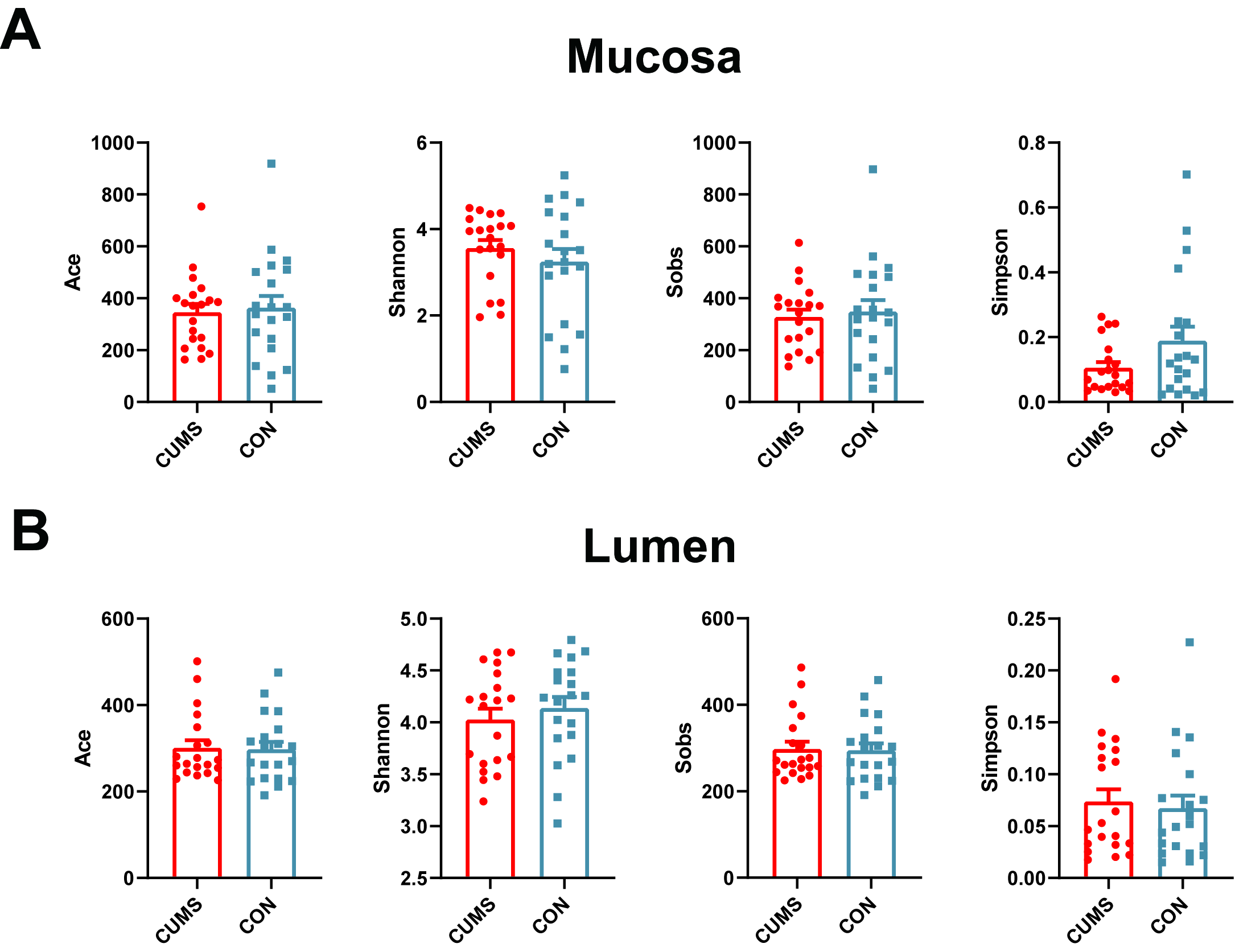

Supplement: Supplementary file 5 — Figure S1 [file 41380_2021_1366_MOESM5_ESM.tif]

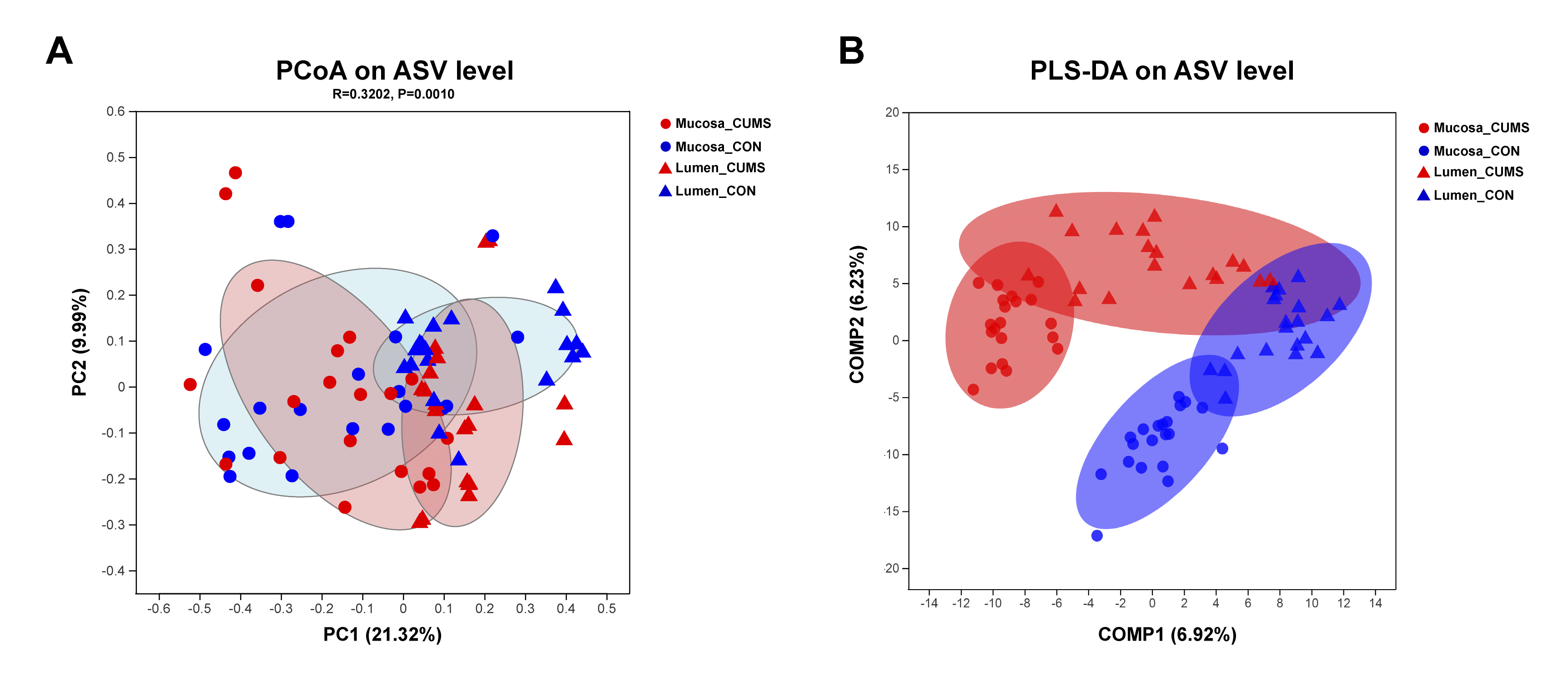

Supplement: Supplementary file 6 — Figure S2 [file 41380_2021_1366_MOESM6_ESM.tif]

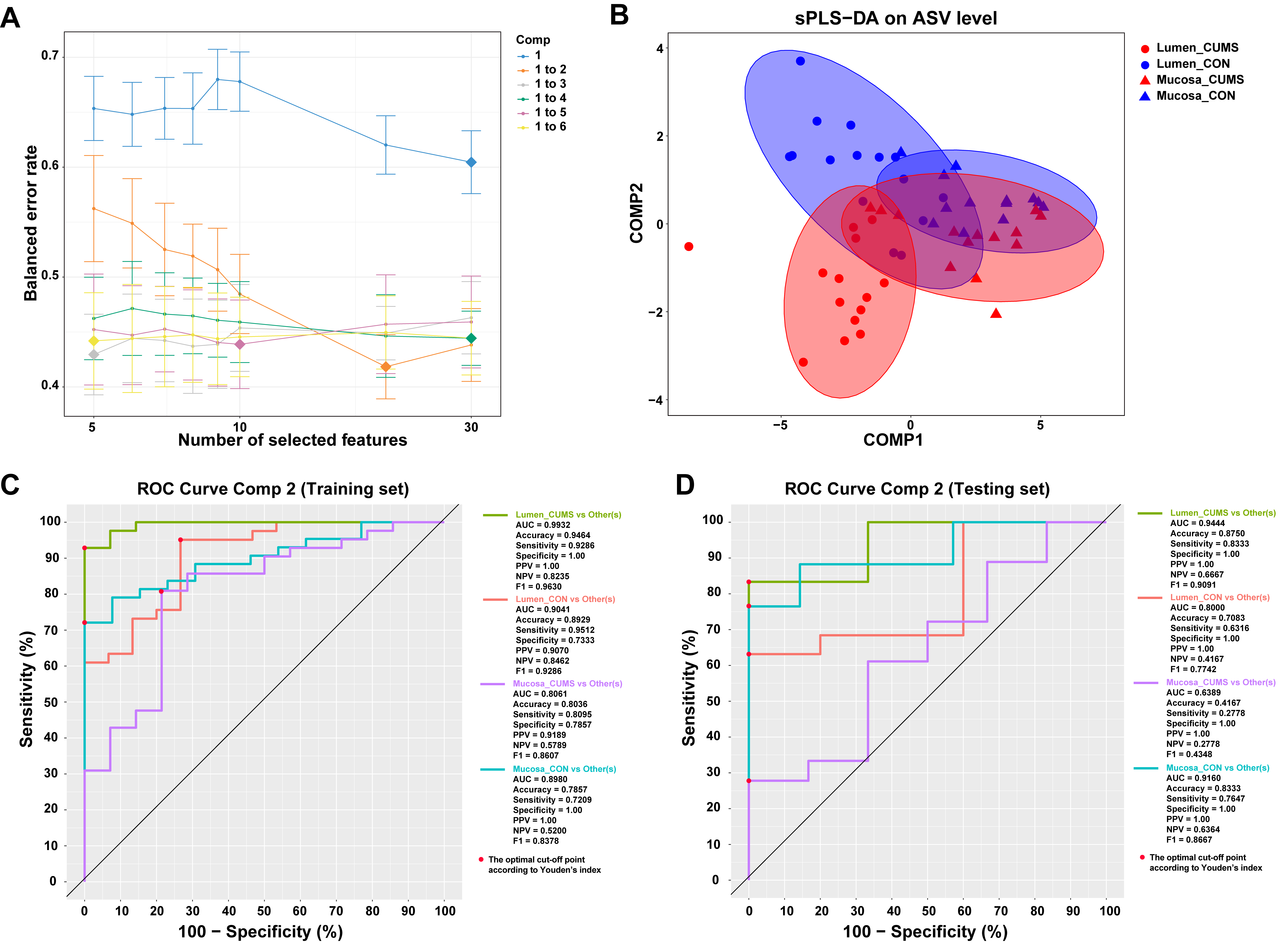

Supplement: Supplementary file 7 — Figure S3 [file 41380_2021_1366_MOESM7_ESM.tif]

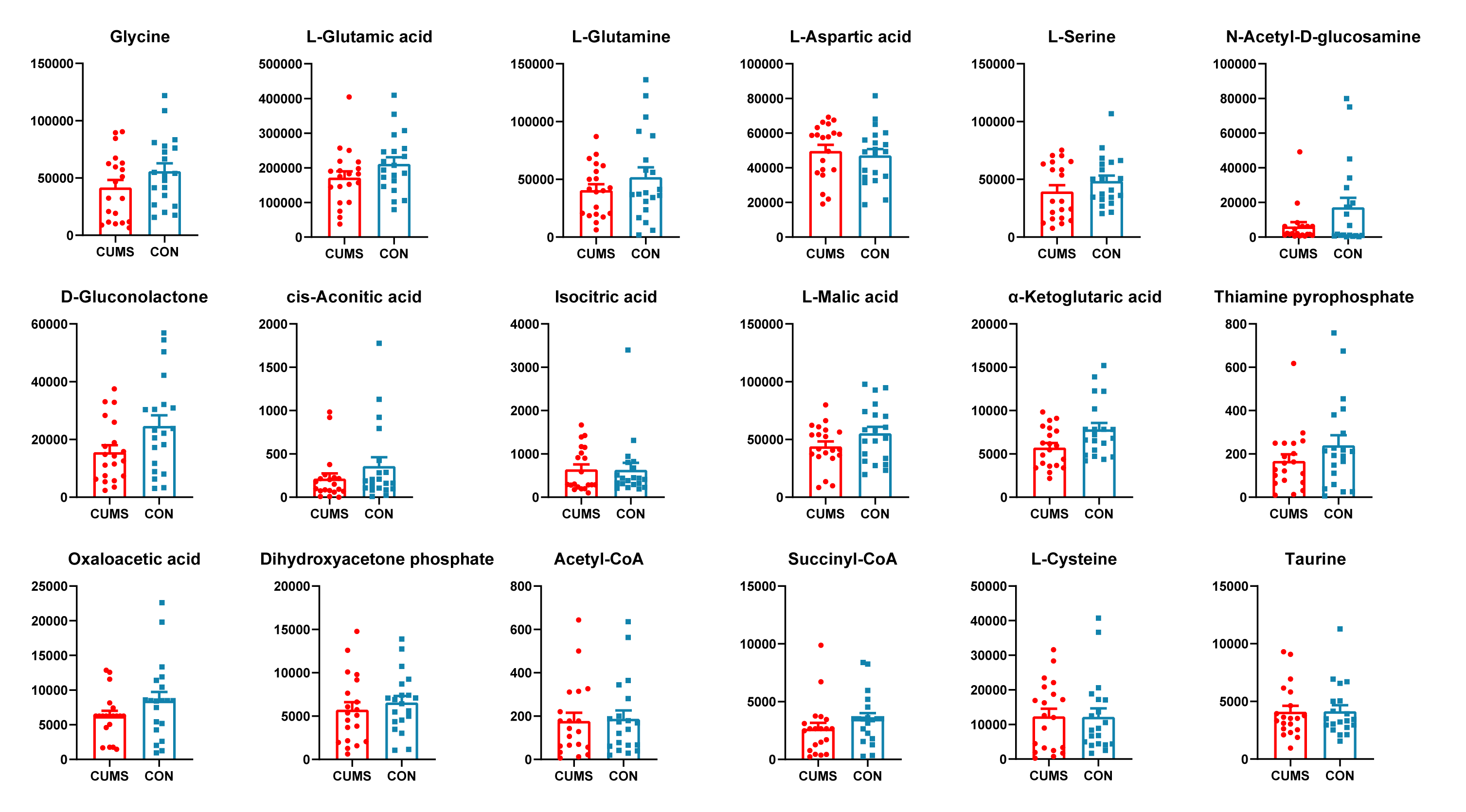

Supplement: Supplementary file 8 — Figure S4 [file 41380_2021_1366_MOESM8_ESM.tif]
